# Forty-year evolution of research topics in the *Journal of Yeungnam Medical Science*: a text mining and keyword network analysis

**DOI:** 10.12701/jyms.2026.43.41

**Published:** 2026-06-21

**Authors:** Seok Hui Kang

**Affiliations:** Division of Nephrology, Department of Internal Medicine, Yeungnam University College of Medicine, Daegu, Korea

**Keywords:** Data management, Data mining, Medical Informatics, Medicine

## Abstract

**Background:**

This study investigated long-term publication trends and thematic evolution in the *Journal of Yeungnam Medical Science* (JYMS) using bibliometric and text mining approaches.

**Methods:**

Publication metadata from JYMS articles published between 1986 and 2025 were analyzed. Author keywords were preprocessed through normalization and stopword removal. Furthermore, keyword frequency analysis, word clouds, trajectory heatmaps, thematic evolution analysis, burst keyword analysis, and keyword co-occurrence network analysis were conducted using Python-based bibliometric methods.

**Results:**

A total of 1,760 articles were analyzed. The average number of keywords per article increased from 1.5±1.7 (1986–1995) to 3.8±1.4 (2016–2025). Earlier decades were characterized by keywords related to pregnancy, psychiatry, and disease-specific topics, whereas recent years demonstrated the increasing prominence of imaging-, metabolic-, and outcome-related research. Coronavirus disease 2019 emerged as the most prominent recent burst keyword. Co-occurrence network analysis demonstrated increasing interdisciplinary connectivity, with imaging-related keywords occupying central network positions.

**Conclusion:**

Over the past four decades, JYMS publications have demonstrated progressive thematic diversification and increased interdisciplinary integration. Bibliometric and text mining analyses may provide valuable information on the journal’s future research directions and academic development.

## Introduction

The rapid expansion of scientific publications has increased the importance of bibliometric and text mining approaches for evaluating research trends and thematic evolution in academic journals. Bibliometric analysis enables the quantitative assessment of publication characteristics, whereas text mining techniques help explore thematic structures and temporal changes in research topics. Collectively, these approaches provide valuable insights into the historical development and future directions of scientific journals [1].

The *Journal of Yeungnam Medical Science * (JYMS) is a multidisciplinary medical journal publishing a broad range of clinical and basic medical research [2]. Since its inception, the journal has progressed with advances in medical science and changes in research priorities. Recently, the journal has increasingly emphasized interdisciplinary integration and global academic visibility [3]. There have been reports on the development of JYMS, encompassing its increasing global profile, indexing status, editorial activities, and publication milestones [3,4]. Park [4] acknowledged the contributions of peer reviewers and the sustained academic growth of the journal. However, these reports are centered on journal performance and editorial perspectives, rather than on the thematic structure and evolution of published research. Therefore, a comprehensive topic-level computational text mining analysis is required.

Although several studies have investigated publication trends in specific medical fields, few have evaluated the long-term thematic evolution of a single multidisciplinary medical journal using computational text mining approaches [5,6]. Understanding these changes may provide insight into the academic identity of the journal and reveal emerging research areas. Previous JYMS reports have focused on journal development and performance, whereas this study mapped research topics using keyword-based text mining and network analysis. Therefore, this study investigated the publication trends and thematic evolution of JYMS publications over the past four decades.

## Methods

**Ethics statement:** Ethical approval was not required for this study because no human participants, human data, or animal subjects were involved.

### 1. Data collection

Publication metadata for articles published in JYMS (1986–2025) were obtained from the Korean Citation Index database. The extracted variables included publication year, author keywords, abstract, and article information. Articles with unavailable publication years or missing keyword information were excluded from keyword-based analyses. Additionally, the publication years were categorized into four periods for temporal analysis: 1986 to 1995, 1996 to 2005, 2006 to 2015, and 2016 to 2025. To assess the standardization and internationalization of author-provided keywords, we obtained official Medical Subject Headings (MeSH) descriptors from the National Library of Medicine and assessed their concordance with the author-provided keywords across different publication periods.

### 2. Text preprocessing and methods

The keywords were converted into lowercase letters and standardized using preprocessing procedures. They were separated using comma- and semicolon-based tokenization. Generic terms with limited thematic relevance, including “study,” “case,” “review,” and “clinical study,” were removed as stopwords. In addition, duplicate keywords within individual articles were removed prior to analysis.

Annual publication numbers were analyzed to evaluate long-term publication trends. Regular and supplementary issues were visualized separately to demonstrate changes in the publication structure over time. Keyword frequencies were calculated for each publication period. Word clouds were generated for each decade using keyword frequencies with larger word sizes representing higher-frequency occurrences. The most frequent keywords from each decade were identified and visualized using bar plots and trajectory heatmaps. Furthermore, a trajectory analysis was performed to evaluate temporal changes in representative keywords across publication periods.

Based on semantic similarity and clinical relevance, keywords were manually categorized into predefined thematic clusters including Imaging/Diagnosis, Oncology, Metabolic/Renal Disease, Surgery/Outcome, Psychiatry/Behavior, COVID-19/Public Health, and Pregnancy/Pediatrics. The representative keywords in each category were identified by reviewing the most frequent author keywords from each publication period. The cumulative frequency of keywords in each thematic category was calculated for each decade. Temporal changes in thematic composition were visualized using a stacked area plot to demonstrate the longitudinal evolution of major research topics across publication periods.

Additionally, burst keyword analysis was performed to identify rapidly emerging research topics across publication periods. Burst scores were calculated by comparing keyword frequencies across consecutive publication periods using the following formula: Burst score=(frequency in the current period+1)/(frequency in the previous period+1), with one added to each frequency to prevent division-by-zero errors. Keywords were classified as burst keywords if they met two criteria: (1) a minimum frequency of three occurrences in the current publication period and (2) a burst score ≥2.0 compared to the preceding period. The burst keywords were then ranked in descending order of burst scores within each publication period. The keywords that showed marked increases during the subsequent publication period were identified as burst keywords.

A keyword co-occurrence analysis was performed using author keywords that appeared within the same publication. Nodes represented keywords, whereas edges indicated the co-occurrence relationships between keyword pairs. The node size was scaled according to the keyword frequency, and the edge thickness indicated the co-occurrence strength. A keyword co-occurrence analysis was performed to map the thematic structure of recent JYMS publications. As the aim was to characterize current keyword relationships rather than temporal changes in network properties, the co-occurrence network analysis was restricted to publications between 2016 and 2025. Only the most frequent keywords and keyword pairs that exceeded a predefined co-occurrence threshold were included in the final network visualization.

### 3. Statistical analysis and visualization

Data preprocessing and visualization were performed using Python (Python Software Foundation, Wilmington, DE, USA). Continuous variables are reported as mean±standard deviation. Bibliometric analyses were conducted using pandas, matplotlib, and NetworkX libraries.

## Results

### 1. Annual publication trends

The average number of keywords per article increased progressively across publication periods. The mean number of keywords per article was 1.5±1.7 from 1986 to 1995, 2.7±1.3 from 1996 to 2005, 3.2±0.9 from 2006 to 2015, and 3.8±1.4 from 2016 to 2025. The total number of published articles was 497, 313, 398, and 552 for each period, respectively. The annual number of publications in JYMS showed an overall increasing trend over the study period (Fig. 1). Publication volume decreased during the early 2000s, followed by a subsequent increase after the mid-2000s. Supplementary issues were also published in 2007 and 2023. The publication peaks observed in 2007 and 2023 may reflect periods of increased output, potentially associated with special issues, editorial initiatives, or other journal-related factors, and should be interpreted with caution.

### 2. Temporal evolution of major keywords

Keyword availability varied across the publication periods. Author keyword availability was 52.1% (259/497) from 1986 to 1995, increasing to 90.7% (284/313) from 1996 to 2005, 99.7% (397/398) from 2006 to 2015, and 96.4% (532/552) from 2016 to 2025. Articles lacking keyword information were excluded from keyword-based analyses but were included in the publication count.

The keyword frequency analysis demonstrated changes in major research topics across publication periods (Fig. 2). From 1986 to 1995, the frequently identified keywords included “pregnancy,” “depression,” “stomach,” and “congenital.” Between 1996 and 2005, keywords such as “myasthenia gravis,” “magnetic resonance imaging,” and “hyperglycemia” appeared frequently. From 2006 to 2015, “hepatocellular carcinoma,” “computed tomography,” and “acute kidney injury” were the most frequent keywords. In the most recent period (2016–2025), “covid-19” was the most frequent keyword. Other commonly identified keywords included “ultrasonography.” The word clouds demonstrated clear temporal changes in research focus across the publication periods (Fig. 3).

Trajectory heatmap analysis using the two most common keywords in each decade demonstrated the progressive evolution of research themes across publication periods (Fig. 4). Furthermore, “ultrasonography,” “depression,” and “hepatocellular carcinoma” demonstrated sustained presence throughout multiple decades, whereas “covid-19” emerged predominantly during recent years. In particular, “ultrasonography” and “covid-19” keywords demonstrated increasing frequency during recent years, reflecting expanding interest in diagnostic imaging and COVID-19-related research. In contrast, several earlier disease-specific keywords such as “myasthenia gravis” and “hyperglycemia” demonstrated reduced prominence over time.

Fig. 4B illustrates the thematic evolution of major research clusters in JYMS publications over the last four decades. During the early publication period (1986–1995), Psychiatry/Behavior- and Pregnancy/Pediatrics-related topics accounted for a relatively large proportion of author keywords, whereas Imaging/Diagnosis and Metabolic/Renal themes were less prominent. From 1996 to 2015, Imaging/Diagnosis-related keywords gradually increased, accompanied by a growth in Oncology and Metabolic/Renal Disease themes. The most prominent thematic expansion was observed between 2016 and 2025. Furthermore, Surgery/Outcome-related themes demonstrated the largest increase in keyword frequency, whereas Imaging/Diagnosis and Metabolic/Renal Disease continued to expand substantially. COVID-19/Public Health-related keywords have emerged during the most recent decade, significantly shaping the overall thematic composition.

The proportion of author keywords that matched official MeSH descriptors increased consistently from 26.6% (1986–1995) to 28.0% (1996–2005), 40.3% (2006–2015), and 62.2% (2016–2025). This progressive increase suggests a growing alignment between author keywords and standardized biomedical terminology, reflecting the journal’s evolution toward internationally recognized indexing practices and greater adherence to controlled vocabulary.

### 3. Burst keyword analysis

Burst keyword analysis demonstrated substantial temporal shifts in research focus across the publication periods. Supplementary Table 1 shows the burst scores. Between 1996 and 2005, “myasthenia gravis” showed notable burst patterns. From 2006 to 2015, the burst keywords primarily included “acute kidney injury” and “rhabdomyolysis.” In the most recent period (2016–2025), keywords such as “covid-19” and “surgery” demonstrated marked burst patterns, suggesting increasing emphasis on COVID-19 in recent JYMS publications.

### 4. Keyword co-occurrence network analysis

The keyword co-occurrence network analysis for 2016 to 2025 revealed several interconnected thematic clusters in recent JYMS publications (Fig. 5). Imaging-related keywords, including “ultrasonography” and “magnetic resonance imaging,” occupied central positions within the network and were closely linked to diagnosis, prognosis, pain, and surgery, whereas rehabilitation and treatment outcome keywords formed another interconnected cluster associated with diabetes mellitus and postoperative complications.

## Discussion

This study investigated long-term publication trends and thematic evolution in JYMS using bibliometric and text mining approaches. Several notable findings emerged from this analysis. First, the overall publication volume demonstrated a gradual increase over the four-decade study period, with a temporary decline in the early 2000s, followed by an increase after the mid-2000s. This pattern may reflect changes in journal operations, research productivity, and subsequent expansion of the journal’s academic activities. The recent publication of supplementary issues further suggests an increase in thematic diversity and academic engagement in the journal. Second, keyword analyses demonstrated substantial temporal changes in research focus across publication periods. During earlier decades, keywords were primarily associated with disease-specific clinical entities and psychiatry- or pregnancy-related topics, including “pregnancy,” “depression,” and “congenital.” In contrast, later publication periods demonstrated increasing prominence of imaging-related keywords such as “computed tomography,” “magnetic resonance imaging,” and “ultrasonography,” highlighting the growing importance of diagnostic imaging technologies in clinical medicine. In addition, topics related to metabolic and renal diseases, including “diabetes mellitus” and “acute kidney injury,” became increasingly prominent in recent decades.

The thematic evolution analysis further demonstrated the progressive diversification of research topics within JYMS. Imaging/Diagnosis-related themes steadily increased across decades, whereas Surgery/Outcome-related topics demonstrated the largest expansion between 2016 and 2025. These findings suggest an increasing emphasis on multidisciplinary clinical research, prognosis evaluation, rehabilitation, and patient-centered outcomes in recent publications. The emergence of COVID-19/Public Health-related themes, exclusively during the most recent decade, indicated the substantial influence of the COVID-19 pandemic on contemporary medical research priorities. The burst keyword analysis identified several emerging period-specific topics. “Myasthenia gravis” demonstrated a significant burst pattern from 1996 to 2005, whereas “acute kidney injury” and “rhabdomyolysis” became prominent from 2006 to 2015. In the most recent decade, “covid-19” demonstrated the most significant burst pattern, reflecting a swift increase in pandemic-related publications. These findings illustrate how research interests in the journal have evolved dynamically with changing clinical and scientific priorities over time.

Additionally, a keyword co-occurrence network analysis characterized the thematic structure of JYMS publications from 2016 to 2025. Imaging-related keywords occupied central network positions, linking diagnosis-, prognosis-, pain-, surgery-, and rehabilitation-related terms, suggesting widespread integration of imaging technologies across multiple clinical domains. Rehabilitation and outcome-related keywords were clustered around diabetes mellitus and postoperative complications, highlighting integrated clinical management and long-term patient outcomes as priorities in recent publications. As the network analyses were limited to recent publication periods, these findings should be interpreted as reflecting the recent research landscape rather than temporal changes in interdisciplinary connectivity.

Another notable finding was the progressive improvement in keyword reporting and standardization over time. Author keyword availability increased substantially over time, rising from 52.1% between 1986 and 1995 to over 96% in recent decades. Furthermore, the proportion of author keywords that matched official MeSH descriptors steadily increased from 26.6% to 62.2% during the study period. These findings suggest that JYMS has progressively adopted standardized biomedical terminology and reporting practices. Structured keywords and controlled vocabulary can enhance article discoverability, indexing accuracy, and information retrieval from bibliographic databases. Studies have shown that carefully selected standardized keywords enhance article discoverability, facilitate literature retrieval, and enhance the visibility and influence of published research [7]. Therefore, improved keyword availability and MeSH concordance may have enhanced the discoverability and international accessibility of JYMS publications, thereby supporting its continued growth and academic visibility.

However, our study has several limitations. First, it was specifically designed to examine the longitudinal thematic evolution of JYMS; therefore, the findings should be interpreted within the context of this journal and may not reflect broader medical publishing trends without comparison to other multidisciplinary or specialty journals. Second, author-provided terminology influenced keyword-based analyses, and variability in keyword selection and standardization may have affected the results despite preprocessing procedures. Third, thematic categorization was performed manually based on semantic similarity and clinical relevance, which might have introduced some degree of subjectivity.

In conclusion, this study provides a comprehensive overview of the longitudinal thematic evolution of JYMS over four decades using computational bibliometric approaches. The findings demonstrate progressive diversification, increased interdisciplinary integration, and the expansion of clinically relevant research topics. They provide valuable insights into the academic development of JYMS and highlight potential research directions for future publications.

## Figures and Tables

**Fig. 1.**
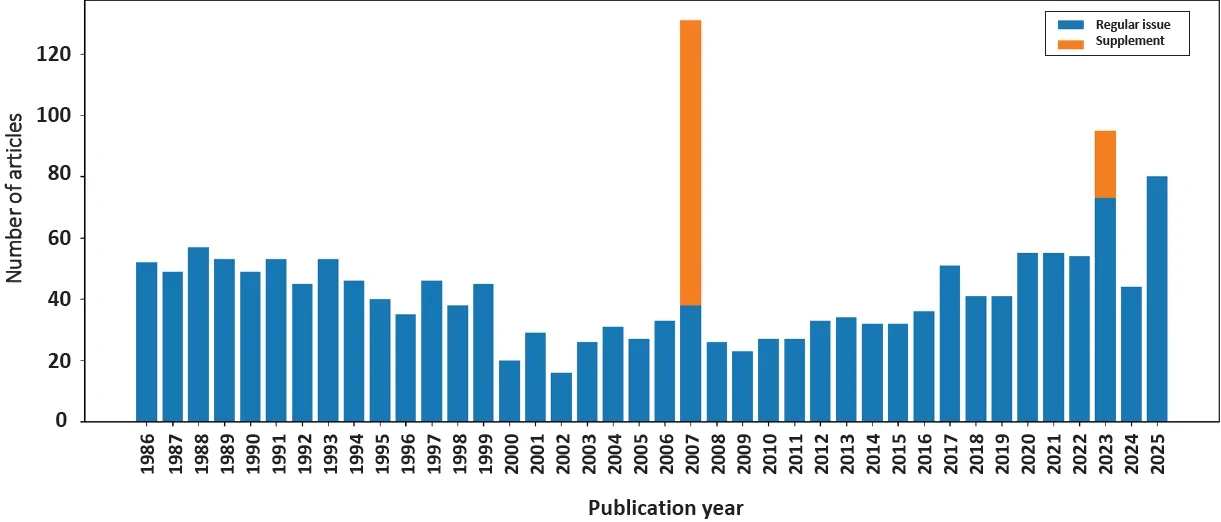
Annual number of publications: regular issues versus supplements.

**Fig. 2.**
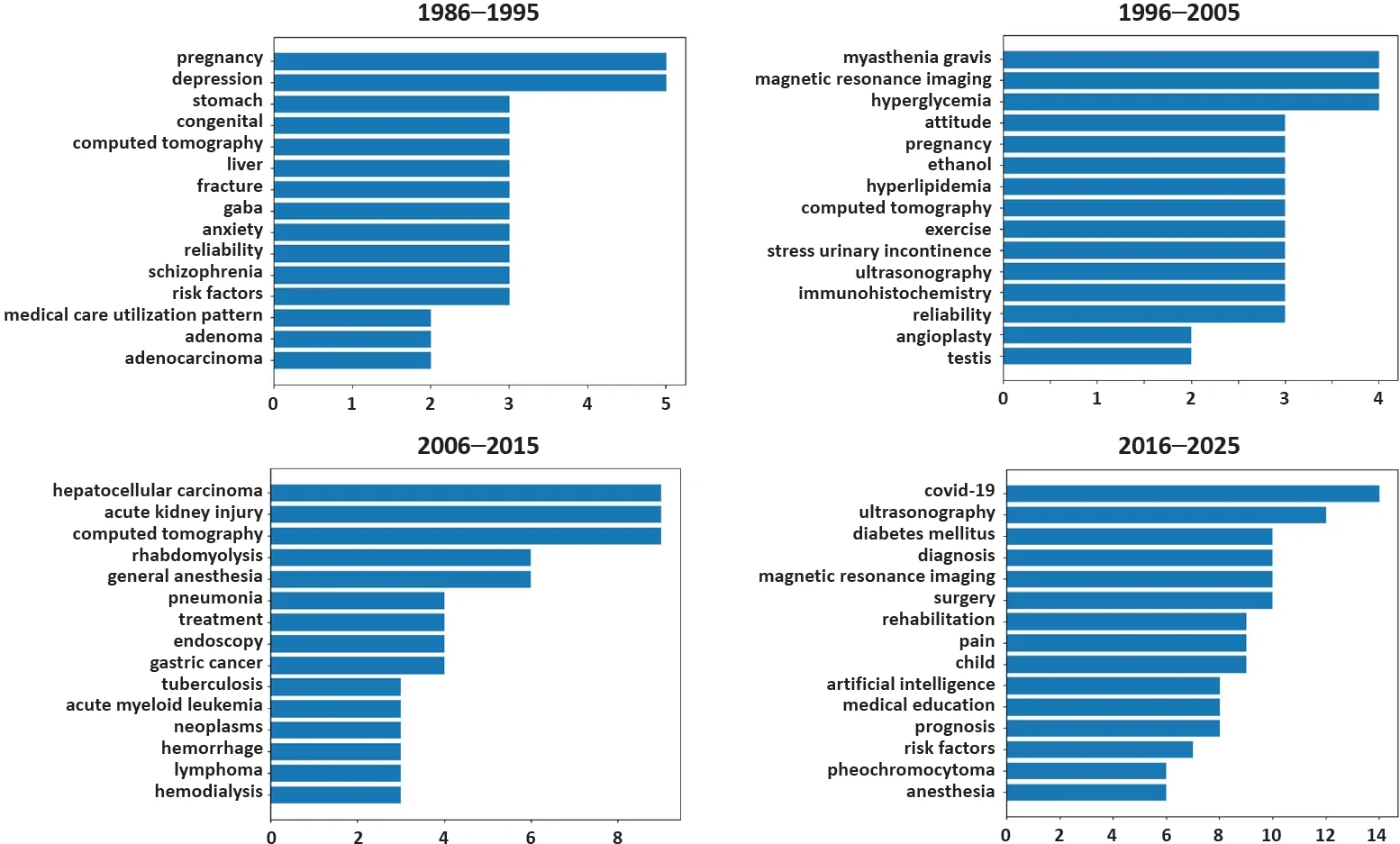
Top keywords by decade.

**Fig. 3.**
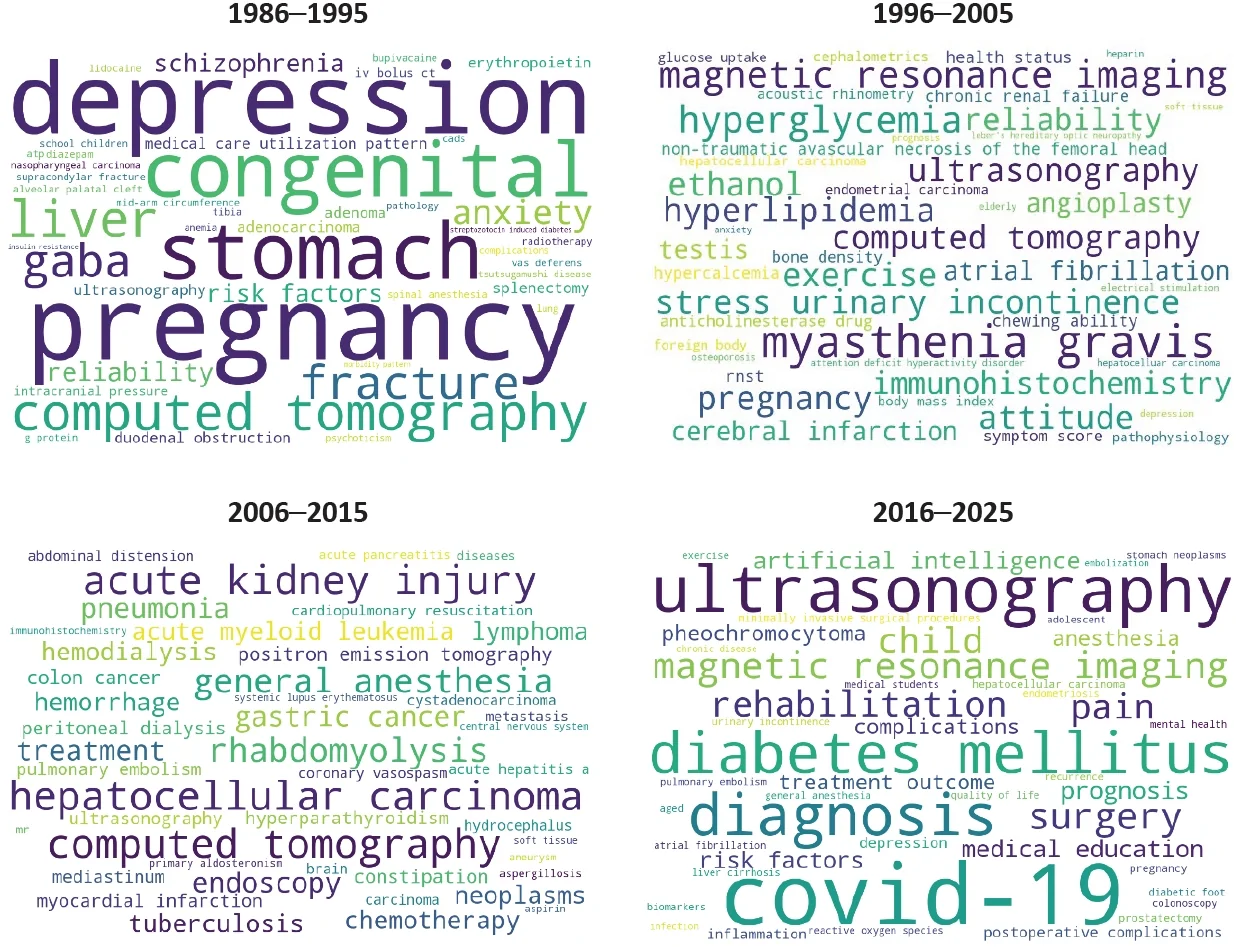
Keyword word clouds by publication period.

**Fig. 4.**
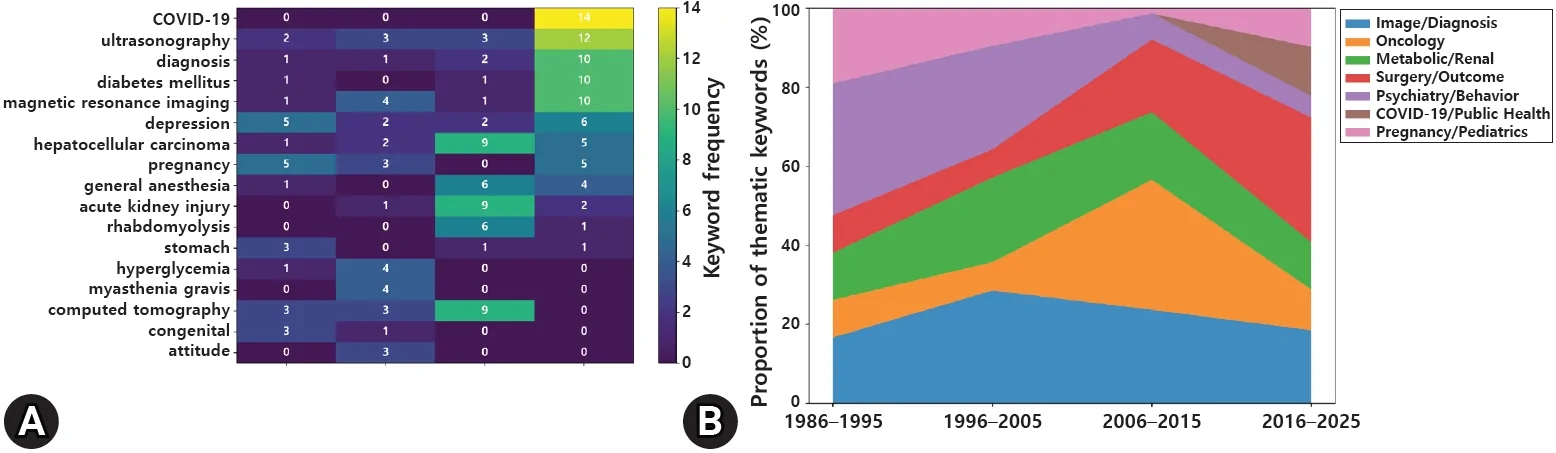
Changes in keywords across publication period. (A) Keyword trajectories. (B) Temporal evolution of major thematic clusters in publications based on keywords.

**Fig. 5.**
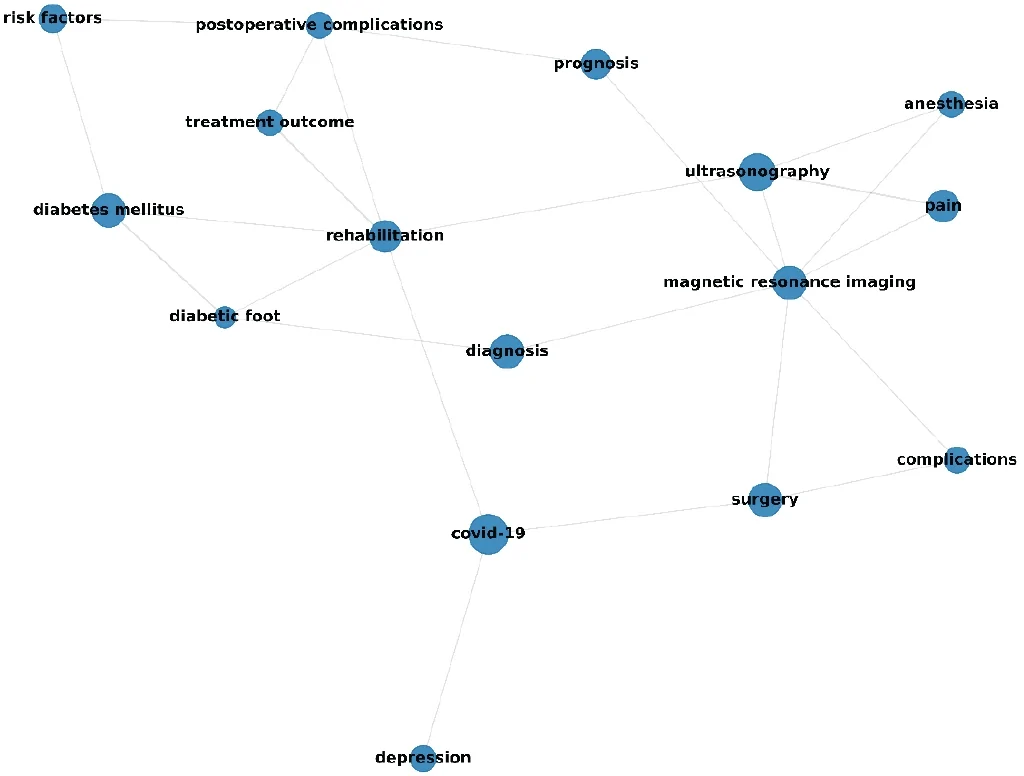
Keyword co-occurrence network analysis of journal publications from 2016 to 2025.

## Supplementary materials

Supplementary Table 1 can be found at https://doi.org/10.12701/jyms.2026.43.41.

Supplementary Table 1. Burst scores across publication period
