# Supplementary material for: Forty-year evolution of research topics in the Journal of Yeungnam Medical Science: a text mining and keyword network analysis
Source: J Yeungnam Med Sci. 2026 Jun 21;43:41. doi: 10.12701/jyms.2026.43.41 (PMC13683986; doi:10.12701/jyms.2026.43.41)
Supplement: Supplementary file 1 [file jyms-2026-43-41-Supplementary-Table-1.pdf]

Supplementary Table 1. Burst scores across publication period

Publication period	Previous period	Keyword	Previous frequency (A)	Current frequency (B)	Burst score
1996–2005	1986–1995	Myasthenia gravis	0	4	5
		Attitude	0	3	4
		Ethanol	0	3	4
		Exercise	0	3	4
		Hyperlipidemia	0	3	4
		Stress urinary incontinence	0	3	4
		Hyperglycemia	1	4	2.5
		Magnetic resonance imaging	1	4	2.5
		Immunohistochemistry	1	3	2
2006–2015	1996–2005	Rhabdomyolysis	0	6	7
		General anesthesia	0	6	7
		Acute kidney injury	1	9	5
		Pneumonia	0	4	5
		Gastric cancer	0	4	5
		Hemorrhage	0	3	4
		Neoplasms	0	3	4
		Myocardial infarction	0	3	4
		Mediastinum	0	3	4
		Acute myeloid leukemia	0	3	4
		Hyperparathyroidism	0	3	4
		Hemodialysis	0	3	4
		Constipation	0	3	4
		Colon cancer	0	3	4
		Chemotherapy	0	3	4
		Tuberculosis	0	3	4
		Hepatocellular carcinoma	2	9	3.3
		Endoscopy	1	4	2.5
		Computed tomography	3	9	2.5
		Treatment	1	4	2.5
		Lymphoma	1	3	2
		Peritoneal dialysis	1	3	2
		Positron emission tomography	1	3	2
		Pulmonary embolism	1	3	2
2016–2025	2006–2015	COVID-19	0	14	15
		Surgery	0	10	11
		Child	0	9	10
		Pain	0	9	10
		Rehabilitation	0	9	10
		Prognosis	0	8	9
		Medical education	0	8	9
		Artificial intelligence	0	8	9
		Postoperative complications	0	6	7
		Treatment outcome	0	6	7
		Prostatectomy	0	5	6
		Minimally invasive surgical procedures	0	5	6
		Inflammation	0	5	6
		Pregnancy	0	5	6
		Magnetic resonance imaging	1	10	5.5
		Diabetes mellitus	1	10	5.5
		Diabetic foot	0	4	5
		Urinary incontinence	0	4	5
		Medical students	0	4	5
		Mental health	0	4	5
		Aged	0	4	5

(Continued to the next page)

Supplementary Table 1. Continued

Publication period	Previous period	Keyword	Previous frequency (A)	Current frequency (B)	Burst score
		Exercise	0	4	5
		Biomarkers	0	4	5
		Stomach neoplasms	0	4	5
		Physiotherapy	0	3	4
		Postoperative pain	0	3	4
		Pediatrics	0	3	4
		Sarcopenia	0	3	4
		Pancreatic cancer	0	3	4
		Neuropathy	0	3	4
		Surveys and questionnaires	0	3	4
		Musculoskeletal pain	0	3	4
		Systematic review	0	3	4
		Lymph node	0	3	4
		Mental disorders	0	3	4
		Meningitis	0	3	4
		Uterine cervical neoplasms	0	3	4
		Pediatric obesity	0	3	4
		Abdominal pain	0	3	4
		Incidence	0	3	4
		Diagnostic imaging	0	3	4
		Cognition	0	3	4
		Hemoptysis	0	3	4
		Chronic disease	0	3	4
		Infection	0	3	4
		Inflammatory bowel diseases	0	3	4
		Drug therapy	0	3	4
		Injections	0	3	4
		Insomnia	0	3	4
		Analgesia	0	3	4
		Large language models	0	3	4
		Diagnosis	2	10	3.7
		Pheochromocytoma	1	6	3.5
		Ultrasonography	3	12	3.3
		Risk factors	2	7	2.7
		Adolescent	1	4	2.5
		Recurrence	1	4	2.5
		Reactive oxygen species	1	4	2.5
		Quality of life	1	4	2.5
		Liver cirrhosis	1	4	2.5
		Endometriosis	1	4	2.5
		Complications	2	6	2.3
		Depression	2	6	2.3
		Anesthesia	2	6	2.3
		Crohn disease	1	3	2
		Stroke	1	3	2
		Venous thrombosis	1	3	2
		Thrombosis	1	3	2
		Differential diagnosis	1	3	2
		Sleep	1	3	2
		Radiotherapy	1	3	2
		Diffuse large B-cell lymphoma	1	3	2
		Dyspnea	1	3	2
		Embolization	1	3	2
		Percutaneous coronary intervention	1	3	2

(Continued to the next page)

Supplementary Table 1. Continued

Publication period	Previous period	Keyword	Previous frequency (A)	Current frequency (B)	Burst score
		Hypertension	1	3	2
		Hyperthyroidism	1	3	2
		Infliximab	1	3	2
		Insulin resistance	1	3	2
		Laparoscopy	1	3	2
		Vertigo	1	3	2

Burst score was calculated as follows: Burst score = (B+1)/(A+1).
